# Distribution and Pathogenicity of Two Cutthroat Trout Virus (CTV) Genotypes in Canada

**DOI:** 10.3390/v13091730

**Published:** 2021-08-31

**Authors:** Amy Long, Francis LeBlanc, Jean-René Arseneau, Nellie Gagne, Katja Einer-Jensen, Jan Lovy, Mark Polinski, Simon Jones, Kyle A Garver

**Affiliations:** 1 Pacific Biological Station, Fisheries and Oceans Canada, Nanaimo, BC V9T 6N7, Canada; amy.long@dfo-mpo.gc.ca (A.L.); Mark.Polinski@dfo-mpo.gc.ca (M.P.); simon.jones@dfo-mpo.gc.ca (S.J.); 2 Gulf Fisheries Centre, Fisheries and Oceans Canada, Moncton, NB E1C 5K4, Canada; Francis.LeBlanc@dfo-mpo.gc.ca (F.L.); Jean-Rene.Arseneau@dfo-mpo.gc.ca (J.-R.A.); Nellie.Gagne@dfo-mpo.gc.ca (N.G.); 3 Qiagen, 8000 Aarhus, Denmark; Katja.Einer@qiagen.com; 4 New Jersey Division of Fish and Wildlife, Office of Fish & Wildlife Health & Forensics, Oxford, NJ 07863, USA; Jan.Lovy@dep.nj.gov

**Keywords:** *Hepeviridae*, *Piscihepevirus*, cutthroat trout virus, CTV-2, Atlantic salmon, Pacific salmon, hepatitis E virus

## Abstract

The sole member of the *Piscihepevirus* genus (family *Hepeviridae*) is cutthroat trout virus (CTV) but recent metatranscriptomic studies have identified numerous fish hepevirus sequences including CTV-2. In the current study, viruses with sequences resembling both CTV and CTV-2 were isolated from salmonids in eastern and western Canada. Phylogenetic analysis of eight full genomes delineated the Canadian CTV isolates into two genotypes (CTV-1 and CTV-2) within the *Piscihepevirus* genus. Hepevirus genomes typically have three open reading frames but an ORF3 counterpart was not predicted in the Canadian CTV isolates. In vitro replication of a CTV-2 isolate produced cytopathic effects in the CHSE-214 cell line with similar amplification efficiency as CTV. Likewise, the morphology of the CTV-2 isolate resembled CTV, yet viral replication caused dilation of the endoplasmic reticulum lumen which was not previously observed. Controlled laboratory studies exposing sockeye (*Oncorhynchus nerka)*, pink (*O. gorbuscha*), and chinook salmon (*O.* *tshawytscha*) to CTV-2 resulted in persistent infections without disease and mortality. Infected Atlantic salmon (*Salmo salar*) and chinook salmon served as hosts and potential reservoirs of CTV-2. The data presented herein provides the first in vitro and in vivo characterization of CTV-2 and reveals greater diversity of piscihepeviruses extending the known host range and geographic distribution of CTV viruses.

## 1. Introduction

Members of the family *Hepeviridae* consist of single-stranded positive sense RNA viral genome encapsidated by a small (typically less than 35 nm in diameter) non-enveloped icosahedral capsid [1]. The *Hepeviridae* family is divided into two genera: *Orthohepevirus* which includes four species that infect humans, birds, bats, and rats, and *Piscihepevirus* which includes one species that infects salmonid fish. In addition to differing in host range, the two genera are phylogenetically distant with cutthroat trout virus (CTV), the sole member of the *Piscihepevirus* genus, being the most distant virus of the *Hepeviridae* family sharing less than 46% nucleotide identity with the Orthohepeviruses [2].

CTV was first isolated in 1988 from apparently healthy cutthroat trout (*Oncorhynchus clarkii*) and has since been isolated in seven different species of trout and Atlantic salmon (*Salmo salar*) [2,3,4]. Despite its non-mammalian host origin, CTV shares genomic similarities with Hepatitis E virus (HEV) that clearly delineate it as a hepevirus. The CTV genome is comparable in size to HEV and maintains three open reading frames as reported for the Orthohepeviruses [3]. Fish either naturally or experimentally infected with CTV have not displayed any gross signs of disease or microscopic pathology suggesting the infection to be non-pathogenic [3,4]. Rather, CTV infections were observed to persist in some host species with viral load often increasing at spawning, possibly due to decreased immunocompetence associated with sexual maturation [2]. The underlying mechanisms responsible for fluctuations in CTV load remain unknown but present parallels with HEV, where infections are generally self-limited, however fulminant hepatitis can occur in immunocompromised patients and pregnant women [5,6], suggesting a possible evolutionary conserved mechanism in the natural history of CTV and HEV. Studies on hepevirus infection/replication, particularly for HEV, were hindered from the lack of suitable in vitro assays due to limited replication of HEV in cell culture. As CTV was found to replicate in an embryo derived cell line, it remains the only hepevirus that can efficiently replicate in cell culture and has shown promise as an HEV surrogate [7,8].

Currently, CTV is the only member officially classified as a *Piscihepevirus*. Nevertheless, through metatranscriptomics, an increasing number of fish hepevirus sequences are being revealed [9] suggesting a broader diversity among the Hepeviridae family. Recent identification of a CTV-like sequence (denoted CTV-2) in salmon sampled from British Columbia (BC) [10,11] (the westernmost province of Canada) suggests greater diversity among CTV virus than presently known. In the study herein, through cell culture screening of salmonids collected from eastern and western provinces of Canada, we isolated viruses with sequences resembling both CTV and CTV-2 demonstrating the widespread nature and diversity of these virus species. Furthermore, we utilized in vitro and in vivo assays to characterize replication, host specificity and pathogenicity of an isolate belonging to the CTV-2 genotype.

## 2. Materials and Methods

### 2.1. Fish Samples and Virus Isolation

Farmed Atlantic salmon, rainbow trout (*Oncorhynchus mykiss*), and Arctic char (*Salvelinus alpinus*) were collected from 2010 to 2017 for research purposes, mortality investigations, or as routine fish health screening as part of the Fish Health Protection Regulations (FHPR) program (Table S1) [12]. Fish were screened for viruses using standard tissue culture methodologies. Tissue homogenates of gill from individual fish or kidney, spleen, pyloric caeca-pancreas, and gills from 5 fish, were inoculated in duplicate onto chinook salmon embryo cells (CHSE-214; ATCC CRL-1681) in 24-well tissue culture plates. Plates were incubated at 15 °C and regularly monitored for cytopathic effect (CPE) for 21 d post-inoculation. If wells showed presumptive CPE, then the supernatant and cells were removed from the well, passed through a 0.45 μm filter, and re-inoculated (undiluted and 1:10 diluted in HBSS) onto newly seeded CHSE-214 and monitored for up to 21 d. Wells displaying CPE were harvested and archived at −80 °C. The virus isolate used for in vivo and in vitro studies, identified as CA/BC/2017-111/AtS, was harvested from CHSE-214 cells 21 days post-inoculum and quantified using the TCID_50_ method as described by Reed and Muench [13].

### 2.2. Virus Sequencing

To identify and characterize the genome of replicating agents observed in cell culture, high-throughput sequencing was performed. For the isolates from eastern Canada, CHSE-214 cells displaying CPE were harvested and total RNA was extracted and purified with the QIAamp UltraSens Virus Kit (Qiagen, Germantown, MD, USA) and Agencourt Ampure XP Beads (Beckman Coulter, Brea, CA, USA). Library construction was then performed using the TruSeq Stranded mRNA Preparation Kit (Illumina Inc., San Diego, CA, USA). The sequencing-ready DNA libraries were submitted to the McGill & Génome Québec Innovation Centre (Montreal, QC, Canada) for paired-end 100 bp sequencing on one HiSeq 2000 lane. De novo contig assembly was done using IDBA-UD (version 1.1.1) and an average of 1095 contigs per sample (length ≥ 200 bp) were obtained. Finally, Geneious (version 9.1.4) was used to map filtered reads to the IDBA-UD generated contigs to obtain whole genome sequences.

For isolates from western Canada, total RNA from virus isolates obtained from two Atlantic salmon netpen sites (four per site) was extracted and purified using Trizol LS, 2 U of DNase I (ThermoFisher Scientific, Waltham, MA, USA,) at 37  °C for 45 min followed by RNeasy MinElute Cleanup (Qiagen) with quality confirmed on a 1% bleach denaturing gel [14]. Library preparation was performed by the Canadian Centre for Computational Genomics at McGill University and Génome Québec Innovation Centre, Montréal, Canada and sequencing was performed on an Illumina HiSeq 2500 (Illumina Inc. San Diego, CA, USA) platform using a NEBNext rRNA Depletion Kit (Human/Mouse/Rat) with read lengths of 125 bp. Each library constituted a 0.125 portion of a sequencing lane. Base calls were made using the Illumina CASAVA pipeline encoded in Phred 33. Data from the two sites were pooled and de novo transcript assembly was performed for each library following the pipeline described by Haas et al. [15] based on the Trinity assembly software suite [16]. Putative assembled transcripts were aligned against the NCBI Viral Genomes Resource database [17] using the blastn program from the NCBI BLAST family.

### 2.3. Phylogenetic Analysis and Genome Annotation

Publicly available sequences were selected to delineate placement of the new Canadian salmonid hepeviruses with previously classified hepevirus taxa (Table S2). Full genome nucleotide sequences were aligned using QIAGEN CLC Genomics Workbench (GWB) 20.0.4 (Available online: https://digitalinsights.qiagen.com/ (accessed on 5 June 2020), using the “very accurate (slow)” alignment parameter settings, with gap costs of 10 for opening a gap and 1 for gap extension. Phylogenetic relationships among isolates were analyzed by using the Create Tree tool, and the neighbor joining approach in combination with the Jukes–Cantor nucleotide distance measure along with 1000 bootstrap replications. Branches were collapsed when the bootstrap values were below 70%. Open reading frames and PXXP motifs were identified using the Find Open Reading Frames tool search function of GWB.

### 2.4. Viral Genotyping on an Expanded Set of Isolates

A panel of 51 isolates collected in eastern Canada between 2010 and 2017 (which vary in host and geographic origin), along with the 8 Canadian CTV isolates and the Heenan Lake 1988 CTV isolate, were aligned and phylogenetically analyzed using a 193 nt region located in the RNA dependent RNA polymerase gene using methods as described in the previous section. Virus RNA was extracted from infected cell culture using Trizol reagent (ThermoFisher Scientific) following the manufacturer’s suggested phenol-chloroform protocol, and glycogen as an RNA carrier. Extracted RNA was reverse-transcribed using the High-Capacity cDNA Reverse Transcription Kit (ThermoFisher Scientific) following the manufacturer’s protocol. The resulting cDNA was then diluted twofold and PCR was carried out using 12.5 µL of AmpliTaq Gold 360 Master Mix (ThermoFisher Scientific), 9.7 µL of DEPC treated water, 0.4 µL of 20 µM forward and reverse primer (Table S3) and 2 µL of diluted cDNA template. PCR was carried out in a T100 Thermal Cycler (BioRad, Hercules, CA, USA) following these conditions: an initial denaturation step at 95 °C for 10 min, 40 amplification cycles of 95 °C for 30 s, 60 °C for 30 s and 72 °C for 30 s, and a final extension step at 72 °C for 7 min. A small volume of PCR product was visualized on a 1% agarose gel for confirmation of an amplified product, and the remaining PCR product was purified using ExoSAP-IT PCR Product Cleanup Reagent (Thermo Fisher Scientific,), following the manufacturer’s protocol. Sanger sequencing was carried out at the McGill University and Genome Québec Innovation Center (Montreal, QC, Canada) using the forward and reverse primers used for PCR. The resulting forward and reverse reads were paired, quality trimmed, and aligned using Geneious Bioinformatics Software for Sequence Data Analysis (version 9.1.8).

### 2.5. Virus Morphology

Electron microscopy was performed on the CTV-2 isolate CA/BC/2017-111/AtS. To obtain sufficient quantities of virus for visualization, the isolate was replicated in four 75 cm^2^ flasks (Thermo Scientific Nunc) of CHSE-214 cells. Cell monolayers were pretreated with 7% polyethylene glycol for 15 min at 15 °C [18] at which time virus was added and incubated for 45 min. Afterwards, 30 mL of fresh MEM-4 and HEPES was added and the flasks were incubated at 15 °C for 12 d to achieve CPE in a minimum of 50% of the cell monolayer. To prepare cells for harvest, 6% glutaraldehyde in 0.1 M sodium cacodylate buffer (pH 7.2) was added to a final concentration of 3% *v*/*v*, and flasks were incubated at room temperature for 1 h. Cells were scraped from the bottom with a tissue scraper. Media and cells from each flask were combined into a 500 mL bottle and 80 mL of the resulting suspension was pelleted by centrifugation (800× *g* for 10 min). Pelleted cells were washed three times with 0.1 M sodium cacodylate buffer, post-fixed in 1% osmium tetroxide in the same buffer for 1 h, washed three more times and then embedded in agar. The agar embedded cell pellets were dehydrated through a graded series of ethanol, cleared in acetone, and embedded in Eponate 12 resin (Ted Pella Inc. Redding, CA, USA). Semi-thin (0.5 µm) sections were cut and stained with Toluidine Blue O stain (Electron Microscopy Sciences, Hatfield, PA, USA) and screened with a light microscope. Five blocks with high concentrations of cells were selected, further trimmed, and ultra-thin (80 nm) sections were cut and mounted on 100-mesh copper grids. Ultra-thin sections were post-stained with 1% uranyl acetate in 50% ethanol for 30 min, washed in distilled water and stained with a modified Sato’s lead stain [19] for 2 min, followed by final washing in distilled water. Samples were viewed using a Philips CM12 transmission electron microscope operated at 80 kV and images taken with a mounted AMT-XR11 digital camera located at the Department of Pathology, Robert Wood Johnson Medical School, Rutgers University. Measurements of virions (*n* = 50) were made directly from digital images taken with magnifications between 35,000 and 45,000×.

### 2.6. CTV-2 Specific Reverse Transcription qPCR Assays

Individual RT-qPCR assays were designed to target either the eastern or western Canada isolates as previously published primers targeting a conserved helicase domain within ORF1 (polyprotein) of CTV [2] were not cross-reactive with isolates in this study. The eastern and western primer and probe combinations were assessed for dimer formation, melting temperature, and product length. Specificities were assessed in silico and cross reactivity of the western Canada CTV-2 assay was empirically tested to ensure no amplification of RNA from Heenan Lake CTV or nucleic acid extracted from Atlantic salmon kidney (ASK) cell line. The limit of reliable detection for the assay was determined from a series of 10-fold dilutions of an artificial positive control (APC) (Integrated DNA Technologies) from 10^5^ to 10^2^ copies/rxn (c/rxn) and fivefold dilutions thereafter to 1 c/rxn. The analytical limit of detection, defined as the concentration in which the APC was detected in >50% of the reactions, was estimated to be 5 c/rxn (Supplemental File 2). The limit of quantification (LOQ) was 54 c/rxn as determined using a curve fitting model with a CV threshold of 35% [20,21] (Supplemental File 2). For the eastern Canada assay, primer specificity was empirically confirmed by endpoint PCR using cDNA from 16 fish species in addition to cDNA from the CHSE-214 cell line.

Total RNA used as template in the specific RT-qPCR assay was extracted from cell cultures using TRIzol LS, gill and kidney tissues using TRIzol Reagent (Life Technologies), and mucus using the MagMAX Viral/Pathogen Nucleic Acid Isolation Kit (Applied Biosystems, Waltham, MA, USA) and Kingfisher Flex (Thermo Fisher Scientific). Tissue was mechanically homogenized with a 5 mm stainless steel bead in a TissueLyser II (Qiagen) for 2 min at 25 Hz. Mucus was homogenized in 1 mL of lysis binding concentrate and spun at 2800× *g* for 5 min. Three hundred microliters of clarified homogenate was added to 700 µL of lysis buffer solution and 20 µL of bead mix into an individual well of a 96 well plate. Plates were then loaded onto the Kingfisher Flex.

RNA was reverse-transcribed using the High Capacity cDNA Reverse Transcription kit (Applied Biosystems). Resulting cDNA was used directly as template for qPCR analysis with a StepOne-Plus real-time detection system using primers and probes optimized for detection of either the isolates from western or eastern Canada (Table S3). Each 25 μL reaction contained 500 nM forward and reverse primer, 250 nM TaqMan probe, 1X TaqMan Universal Master Mix, 2.5 μL cDNA template, and nuclease-free water to reach the final volume. Cycling conditions included an initial incubation of 50 °C for 2 min followed by 95 °C for 10 min, and then 40 cycles of 95 °C for 15 s and 60 °C for 1 min. Samples were assayed in duplicate and were considered positive if both technical replicates had a Ct value < 40 cycles. Absolute quantification was determined by 10-fold serial dilution of the APC ranging from 10^7^ to 10^1^ c/rxn.

### 2.7. In-Vitro Viral Replication

Replication efficiency of the CTV-2 isolate CA/BC/2017-111/AtS was evaluated in CHSE-214, *Epithelioma papulosum cyprini* (EPC), rainbow trout gill (RTgill-W1), and ASK cells. Prior to inoculation onto cells, Atlantic salmon gill homogenate infected with isolate CA/BC/2017-111/AtS was analyzed via RT-qPCR for RNA copy number. Cells were seeded onto 12-well plates and incubated overnight at 20 °C. The following day, cells were inoculated with 100 µL per well of infected Atlantic salmon gill homogenate serially diluted 10-fold from 1.1 × 10^4^ to 1.1 × 10^−3^ c/rxn. For each collection at 14 and 21 d post-inoculation, the entire well was harvested and a 250 µL aliquot, containing the cellular and supernatant fraction, was subsampled for viral load quantification by RT-qPCR. All samples were frozen at −80 °C until needed.

The replication kinetics of the CA/BC/2017-111/AtS isolate was evaluated in RTgill-W1 and CHSE-214 cells. CHSE-214 plates were pretreated with 7% *w/v* PEG as previously described. In 12 well plates, confluent cell monolayers were inoculated with 100 µL per well of gill homogenate and rocked gently for 45 min at 15 °C. As negative controls, equivalent plates were prepared with 100 µL of HBSS added in lieu of virus. After 45 min, 2 mL of media (MEM-4 or ASK) was added to each well and incubated at 15 °C without rocking. Duplicate samples were immediately collected from exposed and negative control plates for the T_0_ sample. Thereafter, samples were collected daily from virus exposed cells and every three days from the negative controls. At time of sampling, an entire well was harvested and total number of cells was enumerated with viable and dead cells differentiated using an Invitrogen Countess Automated Cell Counter. A 250 µL aliquot of the harvested material was subsampled for viral load quantification by RT-qPCR. All samples were frozen at −80 °C until needed.

### 2.8. Disease Challenges

To determine the pathogenicity of the CTV-2 isolate CA/BV/2017-111/AtS to Pacific salmon and evaluate the transmission potential of naturally infected Atlantic salmon, laboratory disease challenges were undertaken at the Pacific Biological Station (PBS; Nanaimo, BC, Canada).

#### 2.8.1. Fish Husbandry

Chinook salmon—*O. tshawytscha* (Qualicum River stock), pink salmon—*O. gorbuscha* (Quinsam River stock), sockeye salmon—*O. nerka* (Pitt River stock) and Atlantic salmon (Mowi-McConnell stock) were acquired from freshwater hatcheries and transported to PBS. Screening of chinook salmon (*n* = 21), pink salmon (*n* = 20), and sockeye salmon (*n* = 11) by RT-qPCR did not detect CTV-2, and no viruses were propagated on CHSE-214 or EPC cell lines. During the pre-screen, CTV-2 was detected in 9/10 Atlantic salmon indicating this population was not naïve to CTV-2.

Fish were reared on EWOS age-appropriate dry pellet rations at 1% body weight per day in 6–8 °C municipal dechlorinated freshwater before being transitioned to sand-filtered, UV-irradiated, 10 °C seawater (salinity 28–32 ppt). Within 1 week of arrival, chinook and pink salmon were transitioned to seawater over a 2-week period. Sockeye salmon were reared in freshwater for 12 months before being transitioned to seawater over a 3-week period. Atlantic salmon were held in brackish water (10 ppt) until the start of the trial when they were transferred to full seawater. All experiments and husbandry practices were carried out with approval of the Pacific Region Animal Care Committee (AUP 19-012 and 19-015) based on guidelines set out by the Canadian Council on Animal Care (CCAC).

#### 2.8.2. Conspecific Cohabitation Trials

To determine if chinook salmon, pink salmon, and sockeye salmon were susceptible to infection with CTV-2 and were capable of transmitting the virus, cohabitation experiments were undertaken in which fish infected with CTV-2 (donor) were cohabitated with an equivalent number of conspecific naïve fish (sentinel) (Table S4). To differentiate the two groups, all donor fish were marked with either floy tags (sockeye and chinook) or had their adipose fin removed (pink). To infect fish with CTV-2, fish were administered an intraperitoneal (ip) injection of isolate CA/BC/2017-111/AtS at a final dose of 10^5.15^ TCID/fish. Fish in the mock infected treatments received an ip injection of an equivalent volume of HBSS. Approximately 24 h post-injection, naïve cohabitants of like species were added to each tank. All tanks were supplied with single-pass, UV-treated seawater at 10 °C (±2 °C) at a minimum flow rate of 1 L per minute per 0.6–0.8 kg of biomass. Fish were monitored daily for up to 18 weeks post-injection (wpi). At 1, 2, 4, 6, 12, 14, 16, and 18 wpi, 3 donor and 3 sentinel were sampled from each of the duplicate treatment and duplicate control tanks and euthanized in tricaine methanesulfonate (MS-222; Syndel Canada). From each fish, mucus and kidney were aseptically collected and flash-frozen in liquid N_2_ for RT-qPCR screening while kidney, spleen, liver, and, heart were preserved in 10% neutral buffered formalin (NBF) for histological examination. Fixation lasted for 24–48 h before samples were dehydrated in an alcohol gradient, clarified in xylene, and infiltrated in paraffin wax. Samples were sectioned at 3 µm, stained with hematoxylin and eosin, and examined by light microscopy. All donor and sentinel fish for each species from 1, 4, 6, 12, 14, and 18 wpi (*n* = 72 fish/species) were examined blinded to treatment group by a board-certified pathologist at the BC Ministry of Agriculture Animal Health Center (Abbotsford, BC, Canada). Lesion severity was assessed on a scale of 0–3.

#### 2.8.3. Heterospecific Cohabitation Trial

As the population of Atlantic salmon was naturally infected with CTV-2, a cohabitation trial was carried out to investigate the transmission potential of this population. Chinook salmon were chosen as naïve recipients as the results of the conspecific cohabitation trials indicated this species was susceptible to CTV-2. Atlantic salmon (*n* = 100, mean weight 90 g) were cohabitated with naïve chinook salmon (*n* = 50, mean weight 76 g) in duplicate 1200 L tanks supplied with single-pass, UV-treated seawater at 10 °C (±2 °C). Fish were monitored daily for 15 weeks post-cohabitation (wpc). Immediately prior to cohabitation and at 2, 4, 6, 10, 12, and 15 wpc, an equal number of Atlantic and chinook salmon (*n* = 5 fish/species) were lethally sampled from each tank. To determine viral load and infection status, kidney was sampled for RT-qPCR analysis as described above.

### 2.9. Statistical Analysis

All CTV-2 positive kidney samples (Ct < 40) were included in infection prevalence calculations as well as in the analysis of pathology data in relation to infection status. Fisher’s exact test of independence (two-sided) was done to test the null hypothesis that detection of CTV-2 by RT-qPCR occurred independently of lesion observation during histopathological examination. Absolute viral copy number (viral load) determined from standard curves was log_10_ transformed prior to analysis. In analyses of viral load data from the heterospecific challenge, only samples with RT-qPCR values above the LOQ were included. A one-way analysis of variance was done to determine if viral load in Atlantic salmon was significantly different between sampling points. A Mann–Whitney–Wilcoxon test was done to determine if differences in viral load between Atlantic salmon mortalities and live sampled fish were significant. All analyses were done in R version 4.04 [22]. For all analyses, results were considered significant if *p* ≤ 0.05. Graphs were prepared in R using the ggplot2 package [23].

## 3. Results

### 3.1. Isolation of CTV-1 and CTV-2

**Figure 1:**
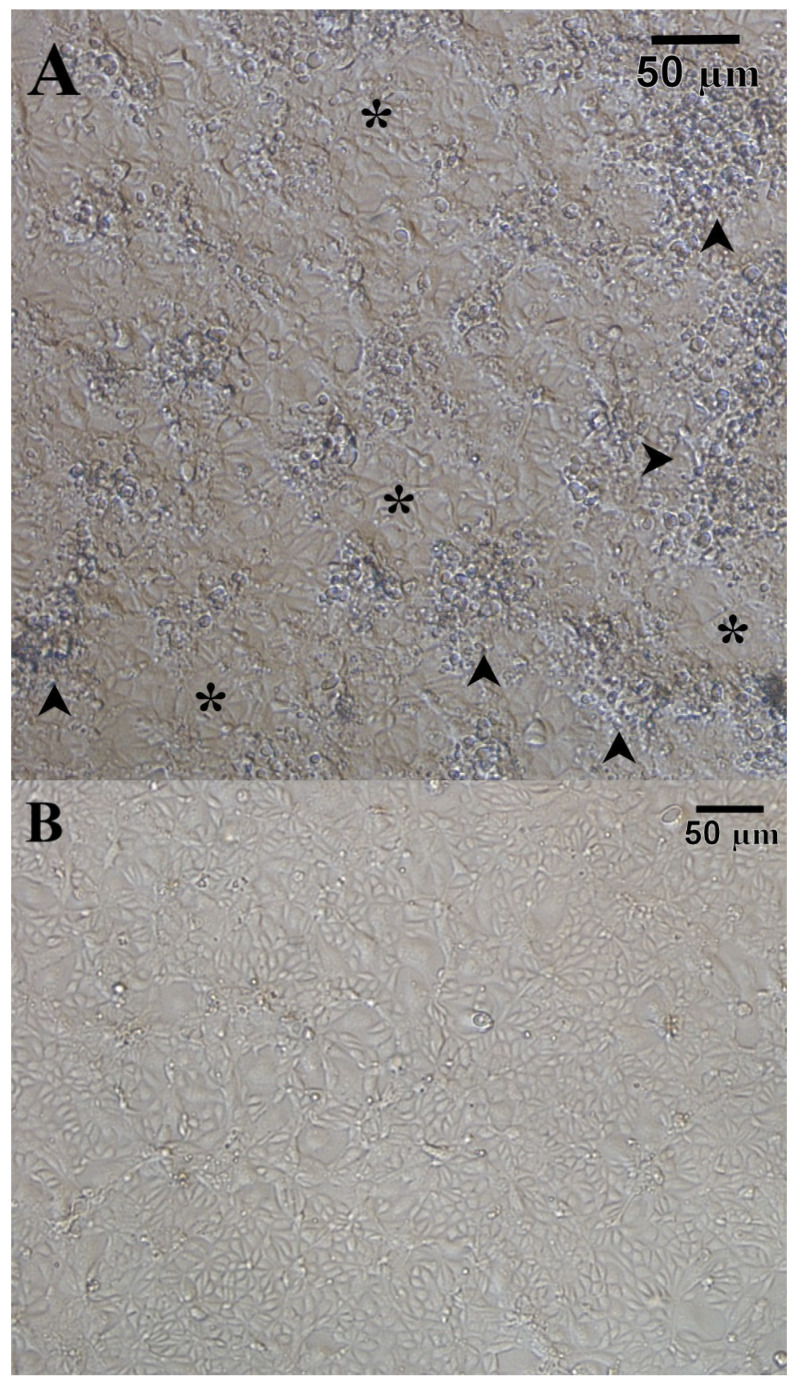
(**A**) Diffuse CPE (arrowheads) and areas of intact monolayer (*) in CHSE-214 cells 5 days after inoculation with CTV-2 isolate CA/BC/2017-111/AtS; **(B)** healthy, uninfected CHSE-214 cells.

CHSE-214 cells inoculated with salmon and trout tissue homogenates containing either gill or a combination of kidney, spleen, pyloric caeca-pancreas, and gills began to show cytopathic effects from 14 to 21 d post-inoculation. The cytopathology was consistent with that reported for CTV [4], namely, restricted to initial focal areas of cell rounding that spread to achieve a diffuse CPE with a ‘foamy’ appearance that does not completely destroy the monolayer (Figure 1). Collectively, 58 virus isolates were obtained from farmed Atlantic salmon in BC and from farmed, hatchery raised or river caught Atlantic salmon, rainbow trout, and Arctic char from provinces in eastern Canada (Quebec, New Brunswick, Nova Scotia, and Prince Edward Island) (Table S1).

### 3.2. Complete Genome Sequence and Phylogenetic Analysis

**Figure 2:**
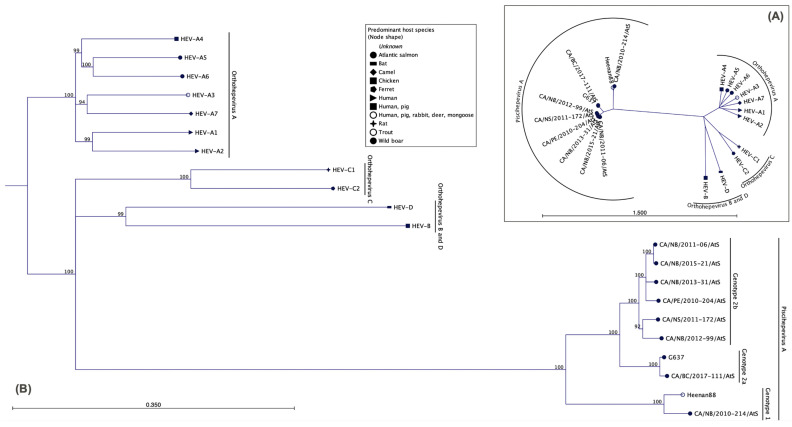
Phylogenetic trees showing the genetic relationship of complete genome sequences of cutthroat trout virus isolates from Canada with other hepevirus isolates (**A**) as radial tree, without bootstrap values); (**B**) as phylogram tree including bootstrap values and the identified sub-genotypes within cutthroat trout virus isolates.

**Figure 3:**
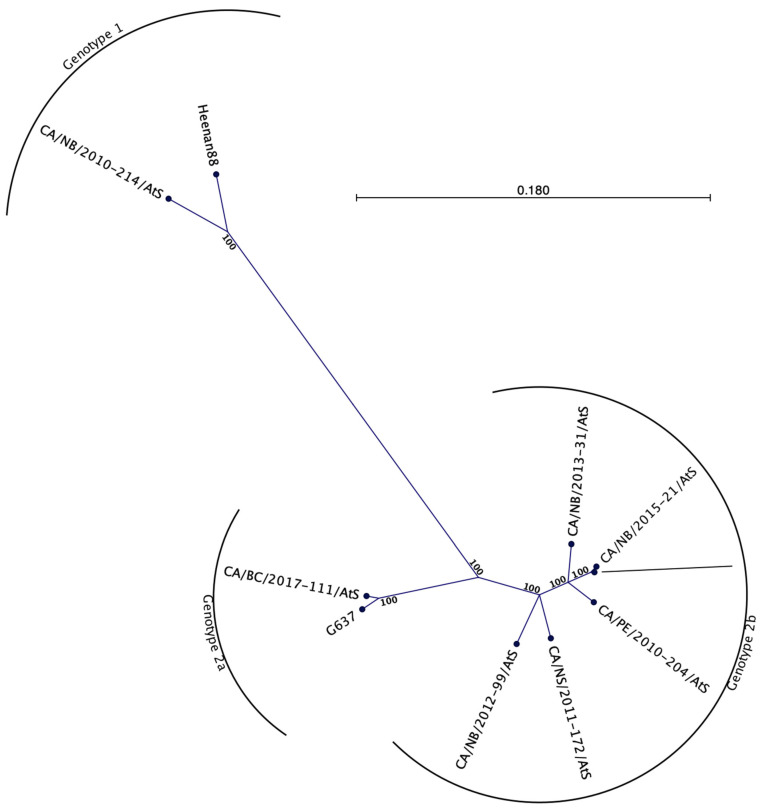
Phylogenetic analysis of members of the *Piscihepevirus* genera, based on complete genome sequences. The isolates cluster into either the CTV-1 or CTV-2 subgenotypes.

The complete genome sequence from eight isolates (7 eastern Canada and 1 western Canada,) ranged from 7127 to 7282 nucleotides (GenBank Accession Number MZ438685–MZ438692) and were between 71.4% and 99.4% identical to one another. Phylogenetically the sequences obtained in our study delineate into the *Piscihepevirus* genus and are clearly genetically separated from the *Orthohepevirus* genus as they share only between 31.6% and 36.4% nucleotide identity between the two genera (Figure 2). Within the *Piscihepevirus* genus, the Canadian sequences were further delineated into two genotypes denoted CTV-1 and CTV-2 (Figure 2B and Figure 3). Genotype CTV-1 includes the isolate previously described from trout from Heenan Lake, California [2], as well as an isolate from Atlantic salmon from New Brunswick described in this study. Genotype CTV-2 consists of two subgenotypes denoted CTV-2a and CTV-2b with subgenotype 2a containing isolates from farmed Atlantic salmon in British Columbia while 2b contains isolates from Atlantic salmon from New Brunswick, Prince Edward Island, and Nova Scotia (Figure 3). Based on full genomes, the maximum number of nucleotide differences between CTV-1 and CTV-2 genotypes was 2109 while the two isolates of genotype CTV-1 differed by 502 nucleotides between each other; among the eight isolates of CTV-2, nucleotide differences ranged from 55 to 996.

Comparison of the cutthroat trout virus sequences in our study to the previously annotated 1988 Heenan Lake CTV isolate and other hepevirus genomes suggests that all Canadian sequences contain open reading frames ORF1 and ORF2 similar to that of other hepeviruses. Within ORF1, non-structural protein domains (including the helicase and RNA-dependent RNA polymerase) were identified with ORF2 predicted to encode the viral capsid protein. However, in contrast to the genome organization of the Heenan Lake CTV and other hepeviruses, a counterpart to the small ORF3 protein was not predicted in the Canadian sequences (Figure S1). A putative protein overlapping reversely with ORF1 was predicted for CA/BC/2017-111/AtS, but not among the other Canadian sequences or the first described CTV genome (Figure S1).

### 3.3. Viral Genotyping on an Expanded Set of Isolates

Phylogenetic analysis based on a 193 nt region of the RNA dependent RNA polymerase gene from 60 isolates differentiated the sequences into two major clades (Figure S2) that mirror the CTV-1 and CTV-2 genotypes assignment established with the restricted set of complete genome sequences. Additionally, the distinction of two subgenotypes within the CTV-2 genotype were maintained with the expanded set of isolates such that CTV-2a is formed by isolates originated from British Columbia while CTV-2b is made up of isolates originated from Prince Edward Island, Nova Scotia, and New Brunswick. Branching structure was observed within CTV-2b, however these groupings did not correlate with either year, location of isolation, or both, and were not supported by strong bootstrap values.

### 3.4. CTV-2 Morphology

**Figure 4:**
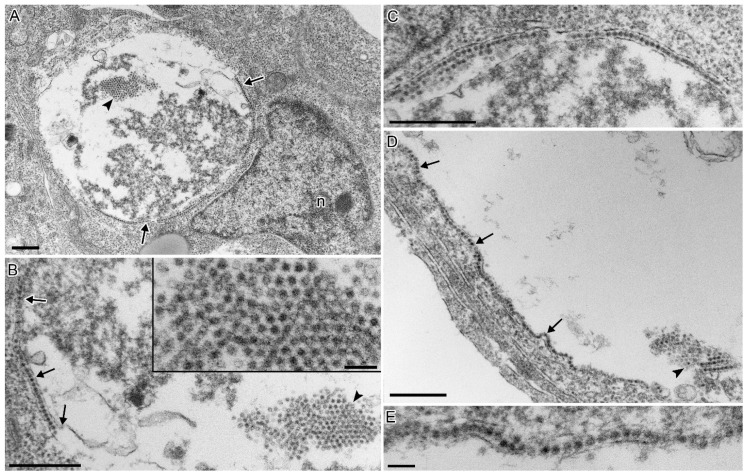
CHSE-214 cells infected with CTV-2. (**A**) A cell with severely dilated ER, making a vacuolar space containing replicating virus. Endoplasmic reticulum (ER) containing viral capsids delimits the vacuole (arrows) and viral capsids are arranged in a crystal array within the vacuole lumen (arrowheads). (n, nucleus) (bar = 500 nm). (**B**) Higher magnification from (**A**) showing viral capsids in a single row within the ER lumen (arrows). Note the inner ER membrane opens to the dilated ER lumen making up the vacuolar space (bottom arrow). Viral capsids arranged in a crystal array (arrowhead and inset) (bar = 500 nm; inset bar = 100 nm). (**C**) Host cell ER, containing one to several rows of viral capsids within the lumen. The ER delimits the host cell cytoplasm (top of image) from the dilated ER lumen containing electron-dense viral proteins (bottom of image) (bar = 500 nm). (**D**) Another infected cell with virions in a single row within the lumen of the ER (arrows) delimiting the host cell cytoplasm and dilated ER lumen containing virions; viral accumulation in a crystal array pattern (arrowhead) (bar = 500 nm). (**E**) Higher magnification from (**D**) showing virions within the lumen of the ER (bar = 100 nm).

**Figure 5:**
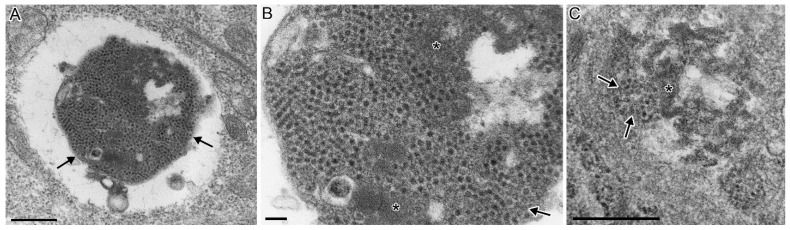
CHSE-214 cells infected with CTV-2. (**A**) dense accumulation of virions (arrows) surrounded by an electron lucent area within the host cell cytoplasm (bar = 500 nm). (**B**) Higher magnification from (**A**) showing viral capsids, empty capsids (arrow) and viral proteins (*) (bar = 100 nm). (**C**) Viral capsids directly within the cytoplasm (arrows) associated with viral proteins (*) (bar = 500 nm).

Virions were non-enveloped, spherical to icosahedral in shape, and had a diameter of 30 ± 1.67 nm (range: 27.6–34.7 nm, *n* = 50). Irregular surface projections occurred from the viral capsid indicating spikes on the surface. The virus occurred only in the cell cytoplasm. The largest viral concentrations were found within the endoplasmic reticulum (ER). The virus occurred within large vacuolated structures within the cytoplasm (Figure 4A), which were continuous with the ER lumens, suggesting that the vacuoles were severely dilated ER. Normal un-dilated ER, composed of two apposed membranes without ribosomes, surrounded and bound the vacuole from the rest of the cytoplasm, with the ER membranes opening to the vacuolar space (Figure 4B). Viral capsids were enclosed and stacked within the ER lumens surrounding the vacuole arranged in a single to multiple rows (Figure 4B,C,E). The severely dilated ER lumen contained viral capsids arranged in a crystal array pattern, along with membranous elements and electron-dense viral proteins (Figure 4A–C). A small number of empty capsids occurred near the normal viral capsids. Other virions occurred which did not appear to be associated with the ER, and this likely reflects different stages in replication. Dense accumulations of virions and viral proteins occurred within the cell cytoplasm (Figure 5A,B,D) within vacuolated structures (Figure 5A) that did not appear to be associated with the ER. In some instances, virions occurred directly within the cell cytoplasm along with viral proteins, which were not enclosed within a vacuole (Figure 5C). A single cell was observed with five enveloped virus-like particles which were outside the plasma membrane. This appeared to be a rare finding, as it was not observed in additional cells.

### 3.5. In Vitro Viral Replication Efficiency and Kinetics

**Figure 6:**
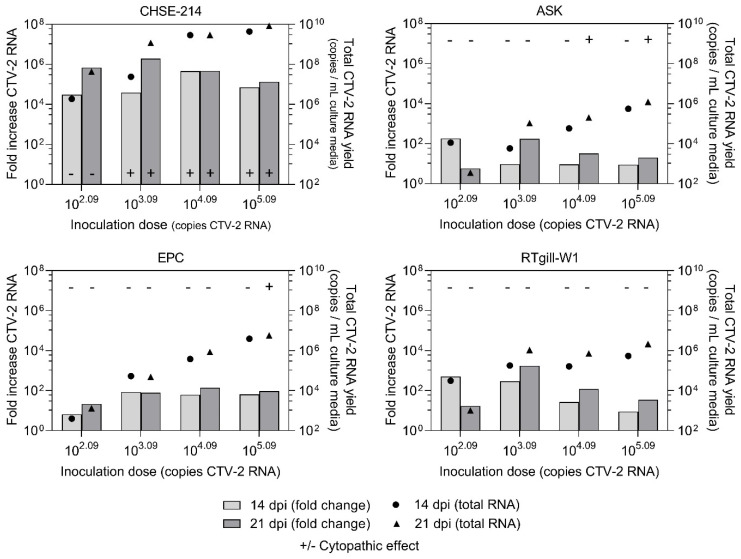
Quantification of CTV-2 (copies/mL culture media) in ASK, CHSE-214, EPC, and RTgill-W1 cells at 14 and 21 d post-inoculation. Plus sign denotes cytopathic effect, which was observed for that concentration at the given timepoint.

**Figure 7:**
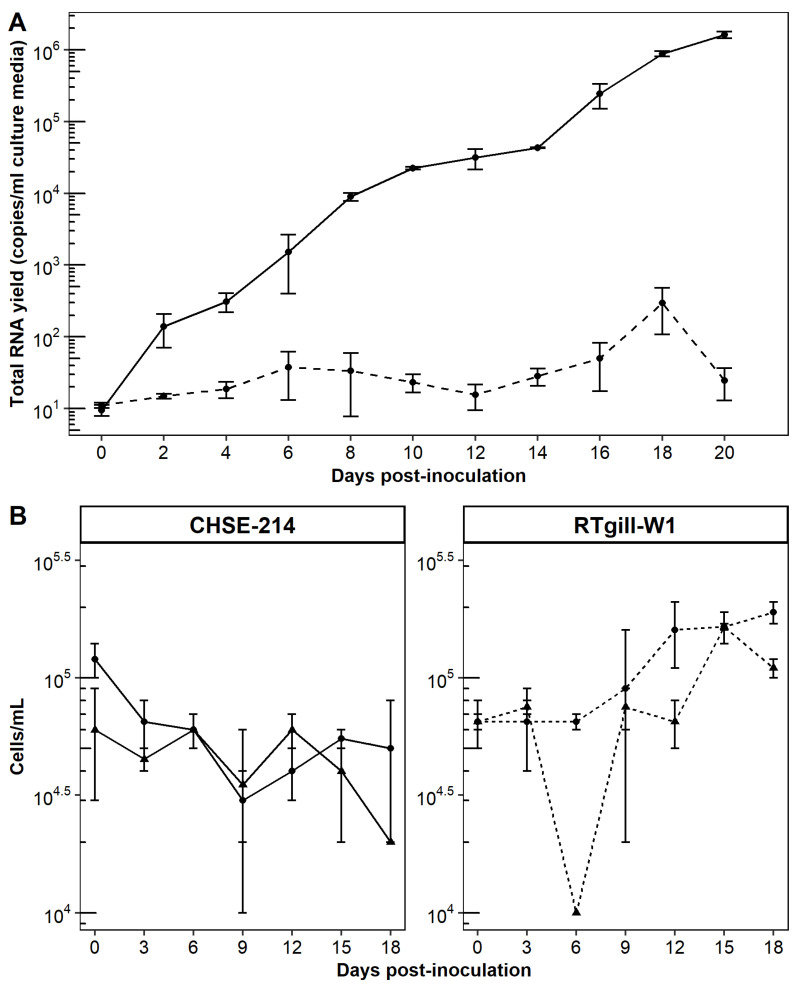
(**A**) CTV-2 replication in CHSE-214 (**-**) and RTgill-W1 (---) cells. Error bars denote standard error of the mean for duplicate samples. (**B**) Viable cells/mL in infected (●) and uninfected (▲) CHSE and RT-gill cells.

Replication of the CTV-2 isolate was best supported in the CHSE-214 cell line with increases in viral load of four to six orders of magnitude. Among the cell lines tested, CHSE-214 was also the only cell line to consistently exhibit CPE associated with the hepevirus infection. Conversely, the ASK, EPC, and RTgill-W1 cell lines were largely absent of CPE and increases of CTV-2 RNA loads greater than two orders of magnitude were rare (Figure 6). Nevertheless, increases in CTV-2 RNA were evident across all cell lines tested. Time course sampling demonstrated CHSE-214 cells permitted continuous replication over a 20-day period increasing from 1.0 c/mL cell culture supernatant (log_10_) at T_0_ to 6.2 c/mL cell culture supernatant at T_20_ while viral replication in RTgill-W1 cells was limited over the 20-day period with an increase in RNA load from 1.0 c/mL cell culture supernatant at T_0_ to 1.3 c/mL cell culture supernatant at T_20_ (Figure 7A). To confirm that infection alone did not decrease the number of viable cells, thereby impacting viral load, the number of viable cells was measured in both infected and uninfected CHSE-214 and RTgill-W1 cells. The number of viable cells remained constant in both cell lines between T_0_ and T_18_ (Figure 7B).

### 3.6. Fish Exposures

#### 3.6.1. Conspecific Cohabitation Challenge

**Figure 8:**
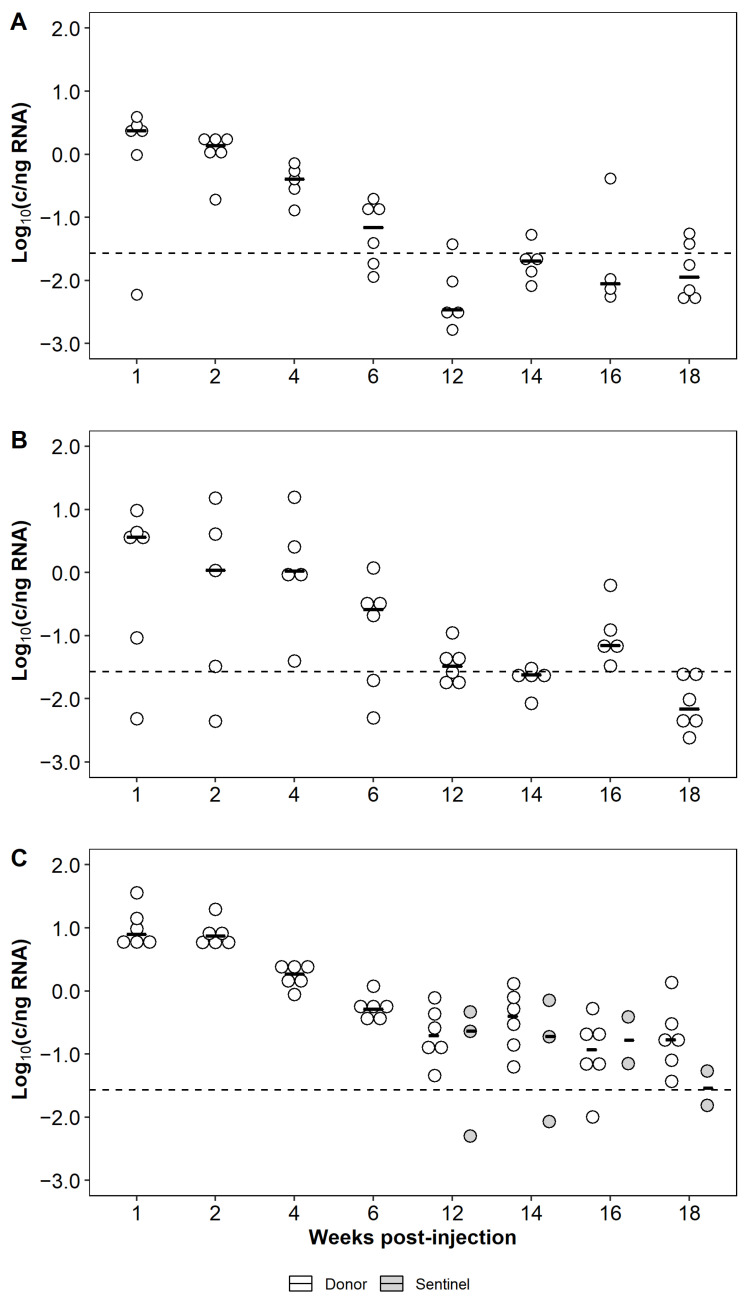
CTV-2 load (log_10_ copies/ng RNA) and prevalence in kidney of (**A**) sockeye, (**B**) pink, and (**C**) chinook salmon in the conspecific cohabitation trial. Six fish were sampled per treatment at each time point and each dot indicates a positive fish. Crossbars denote median viral load. The dashed line denotes the reliable limit of detection of the qPCR assay.

Among the donor sockeye, pink, and chinook salmon, virus was detected in kidney from each species over the entire 18 week sampling period, albeit with varying prevalence and intensity among species (Figure 8). Post-injection, all species of donor salmon demonstrated a successive decline in the median viral kidney loads during the first 12 weeks of sampling after which Chinook median loads remained relatively consistent while Sockeye and Pink salmon median viral loads occurred at or below the reliable detection limit of the RT-qPCR.

For the sentinel cohabitant species, only chinook salmon acquired internal infections (Figure 8). Kidney infections of sentinel chinook salmon were first detected at 12 wpi and continued to the completion of the study at 18 wpi during which prevalence ranged from 33% to 50% (Figure 8). Sentinel pink and sockeye salmon did not acquire kidney infections, yet the presence of CTV-2 in mucus swabs from pink, sockeye, and chinook salmon sentinels within the first four weeks of sampling (Table S5) suggests all sentinel species were indeed exposed to the virus.

No mortality or morbidity specific to the CTV-2 treatment group occurred during the 18-week study. One donor sockeye salmon died from handling injuries two days after the start of the study and one moribund sentinel chinook salmon (negative for CTV-2 by RT-qPCR) with tail rot was euthanized 7 wpi. Among the pink salmon, failure to feed after seawater transition (pinheading) resulted in 7% average mortality regardless of the treatment accounting for a total of 10 mortalities in the negative control tanks (*n* = 4, *n* = 6) and 12 in the CTV-2-exposed tanks (*n* = 6, *n* = 6). Histopathological examination of liver, kidney, spleen, and heart tissue from donor and sentinel fish of all species at 1, 4, 6, 12, 14, and 18 wpi (Supplemental File 3) did not reveal substantive or sustained pathology in relation to CTV-2 infection. There was no association between CTV-*2* detection and lesion observation with the exception of mild endocarditis in chinook salmon (*p* = 0.035) and intratubular protein casts in sockeye (*p* = 0.017) and chinook (*p* = 0.004) salmon (Supplemental File 3). Pink salmon had no discernible difference in pathology between individuals infected with CTV-2 versus those without detectable CTV-2.

#### 3.6.2. Heterospecific Cohabitation Challenge

**Figure 9:**
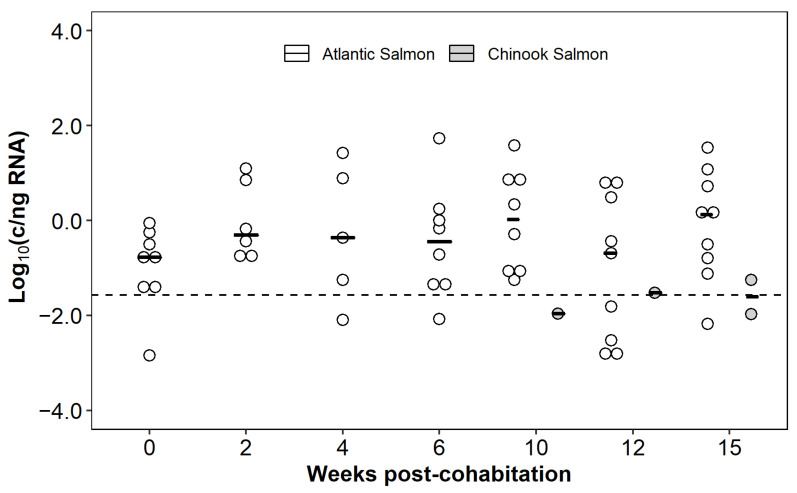
CTV-2 load (log_10_ copies/ng RNA) and prevalence in donor Atlantic salmon and sentinel chinook salmon kidney from the heterospecific cohabitation trial. Ten fish were sampled at each time point and each dot indicates a positive fish. Crossbars denote the median viral load at each sampling time. The dashed line denotes the reliable limit of detection of the qPCR assay.

To determine if Atlantic salmon naturally infected with CTV-2 could transmit the virus to naïve chinook salmon, the species were cohabitated for 15 weeks. During the first six weeks of sampling, chinook salmon remained free of CTV-2, however by 10 wpc, CTV-2 was detected in the kidney of one chinook salmon. Subsequent detection of CTV-2 at 12 and 15 weeks confirmed virus transmission to the naïve chinook salmon (Figure 9). No signs of disease or significant mortality were observed among the chinook salmon over the course of the study. One chinook salmon died at 2 wpc, however CTV-2 was not detected in the kidney of this fish.

In CTV-2-infected Atlantic salmon, virus was consistently detected in kidney sampled over the 15-week study with prevalence reaching upwards of 90% (Figure 9). Viral load in kidney remained relatively constant throughout the challenge (Figure 9) with no significant difference observed between any time points (F_6,22_ = 0.64, *p* = 0.7). Fifteen Atlantic salmon died during the 15-week study with a mean day to death of 44.5. Due to the lack of an uninfected Atlantic salmon control group, the role of CTV-2 in these mortalities is uncertain. Prevalence of CTV-2 in the mortalities was 87% (Supplemental File 2). The median load of CTV-2 was significantly different between the live sampled fish and those that died (Figure S3; 0.33 vs. 1.2 log_10_ c/ng RNA, *p* = 0.045).

## 4. Discussion

The detection of CTV in eastern and western Canada reveals a wider distribution, extended host range, and greater diversity of the *Piscihepevirus A* species of viruses than presently known. With the exception of a partial sequence resembling CTV from Atlantic salmon in New Brunswick, which was initially described as a togavirus-like virus [24], CTV has predominately been considered a virus infecting trout within various watersheds of the western United States and to date, CTV-2 was only described in chinook and Atlantic salmon from British Columbia [10,11]. However, from the isolation of CTV-1 and CTV-2 from rainbow trout, Atlantic salmon, and Arctic char sampled from Quebec, Nova Scotia, Prince Edward Island, and New Brunswick, it is clear these viruses extend beyond the geographic range of western North America and are viruses of salmonid fishes belonging to the genera of *Salmo*, *Oncorhynchus*, and *Salvelinus*.

Phylogenetic analysis based on either full genome or partial gene sequences of Canadian CTV viruses differentiated the isolates into two distinct genotypes. This genetic delineation of CTV viruses does not appear to correlate with geographic separation as both CTV genotypes co-circulate in eastern and western regions of North America. In particular, the high sequence identity of CTV-1 isolates from areas of western North America and eastern Canada suggests a continued shared virus origin despite being geographically separated. Despite sharing a common ancestor, it is notable that genetic divergence is apparent between isolates from eastern and western Canada, suggesting that CTV-1 and CTV-2 are likely under different evolutionary constraints. The exact source(s), mechanism(s), or both, responsible for widespread distribution of CTV genotypes remains unclear, however, vertical transmission has been speculated [3] given the detection of CTV-1 in 1 month old offspring of virus-positive broodstock [4]. Currently, we are conducting laboratory investigations to determine whether a CTV-2 isolate can be spread via intra-ovum transmission with the overarching goal of providing insights into how these viruses are maintained in salmonid populations.

The morphology of the CTV-2 isolate is consistent with CTV as well as with other hepeviruses, which are defined as 27–34 nm, non-enveloped virions with spikes on their surface [25]. The findings of CTV-2 directly within the host endoplasmic reticulum (ER) lumen demonstrates the importance of ER in the replication of hepeviruses. Though the general lack of an in vitro culture system has limited the understanding of hepevirus replication, the collective literature shows that HEV, a related Orthohepevirus, replicates within the ER as reviewed by [26]. A unique finding with CTV-2 was the pattern of viral replication causing severe dilation of the ER lumen producing a large vacuole containing viral capsids. The periphery of the vacuole lumens with un-dilated ER containing one to several rows of viral capsids. The pathologic modification of the ER during viral replication should be further elucidated to understand viral replication and the resulting cellular pathology related to CTV, which may improve the basic understanding of hepevirus replication.

Following development of viral capsids, it has been reported that HEV virions acquire a quasi-envelope following egress from multivesicular bodies in the exosomal pathway [27,28]. Similarly, CTV derived from cell culture was demonstrated to be lipid-associated [7] and shared the characteristic quasi-envelope after being released into the cell culture [8]. The mechanism for the release of hepeviruses from infected cells is not well characterized, however the ORF3 protein is thought to be instrumental in this process. ORF3 of HEV forms multimeric complexes that associate with intracellular ER-derived membranes and act as a virally encoded ion-channel to facilitate release of infectious particles [29]. Additionally, a PSAP motif, conserved across ORF3 proteins of all known HEV strains, including avian HEV, appears necessary for the formation of membrane-associated HEV particles [30,31,32,33]. The involvement of ORF3 in the egress of particles is interesting to consider in the context of CTV-2, as the particular isolate evaluated in our study appears to be lacking a putative homolog of ORF3 which was previously reported for a CTV-2 isolate from BC [10]. A PTAP motif (Table S6), along with a prominent serine rich region (22–28 amino acids; Figure S4), was identified within the non-structural polyprotein of the Canadian Piscihepeviruses; however, it is unknown whether these sites have any functional involvement in the release of virus. A rare observation of CTV-2 (isolate CA/BC/2017-111/AtS) enveloped virions outside the cell plasma membrane (results not shown) suggests that CTV-2 shares the capacity to acquire a quasi-envelope as demonstrated for CTV and HEV [8], though the origin of the envelope in CTV-2 would require further elucidation. Nevertheless, as the CTV-2 infected cells observed in our study were fixed for TEM at a single time point post infection, additional studies are required to evaluate CTV-2 across all stages of viral replication, assembly, and egress in an effort to compare with the stages observed for CTV and HEV.

In cell culture, CTV-2 produced CPE and replicated in CHSE-214 cells with an efficiency similar to that described for CTV, where viral growth of approximately four orders of magnitude was measured within two weeks [34]. CTV-2 also resembled CTV in that both genotypes failed to replicate to high levels in the EPC cell line. However, our observation of minimal CTV-2 propagation in RT-gillW1 cells is in contrast to what was reported for CTV which replicated to high titers within short incubation times [7]. Such differences in propagation between CTV genotypes may not truly be a result of viral strain differences, but rather a consequence of differences in methodology and cell origin between laboratories. Alternatively, this differential growth of CTV and the CTV-2 isolate in rainbow trout cells may reflect a genotype-associated host specificity. However, it is worthwhile to note that such host specificity does not appear to be mirrored in vivo such that detections of CTV-2 were made in various trout species including rainbow trout (William Batts, Western Fish Research Center, United States Geological Survey, personal communication), indicating that the two CTV genotypes overlap in host range with each maintaining the capacity to infect both salmon and trout. Whether or not either genotype exhibits a preference or adaption for infecting a specific host species will require additional research specifically involving side by side comparisons of the two genotypes.

Based on our controlled laboratory exposure study with Pacific salmon, infections with CTV-2 appeared to be long-lasting with detectable levels of viral RNA present in donor fish throughout the 18-week study. Nevertheless, not all CTV-2 persistent infections were communicable. The inability of CTV-2 infected pink and sockeye salmon to pass virus to sentinel cohabitants suggests that these species are unlikely to serve as either a natural host or vector of CTV-2. In contrast, chinook salmon were susceptible to CTV-2 infection resulting in conspecific and heterospecific transmission thereby revealing their ability to serve as a host, reservoir, and/or biological vector of CTV-2. Regardless of exposure route, CTV-2 infections in Pacific salmon did not result in mortality, disease, or substantive pathology during the 18 week study. Although CTV-2 infected chinook salmon had a higher proportion of mild endocarditis, we hypothesize this mild inflammation is not a specific response to CTV-2 but rather a generic physiological response to antigens (calf serum, antibiotics) present in the virus inoculum which was intraperitoneally injected into donor fish. This is supported by a higher prevalence of mild endocarditis in donor fish at 1 wpi which decreased over time coinciding with a steady decline in virus load (Supplemental File 3). Donor chinook and sockeye salmon also had increased formation of intratubular protein casts (IPC) in kidney tissue. It should be noted that while there were three instances of moderate IPC in positive chinook salmon donors, there was also a moderate IPC in a negative sockeye salmon sentinel. Additionally, none of the positive chinook salmon sentinels developed moderate IPC suggesting that factors other than CTV-2 may be influencing the accumulation of renal tubular protein.

Overall the pathology observed in Pacific salmon infected with CTV-2 was mild, and even among those with a moderate score, there was no evidence these changes had a significant effect on the overall health of the fish. Nevertheless, the fate of hepevirus infections are intimately linked to the host’s condition and associated immunological responses. In humans, the hepatitis E virus is one of the most common causes of acute hepatitis, typically resulting in mild self-resolving infections. Yet in pregnant women with HEV, mortality is increased dramatically, a phenomenon thought to be a consequence of hormone induced immunological changes associated with pregnancy [35,36,37]. Furthermore, although CTV infections of salmonids are nonpathogenic, increased viral replication and shedding during spawning has been demonstrated [2]. In fact, the modulation of CTV replication through the experimental application of various sex steroids to fish cells [34] illustrates similarities in underlying mechanisms affecting CTV and HEV replication. Interestingly, the higher loads of CTV-2 in Atlantic salmon mortalities as compared to live fish as described herein and observed by Bateman et al. [11] for two of four farm cohorts, may be an indicator of immune modulation of CTV-2, whereby persistent infections are intensified in compromised hosts. Further research to elucidate factors responsible for pro/antiviral effects on CTV viruses are needed and are underway in our laboratory. As salmon and trout can accommodate mixed etiological infections, it is of particular interest to evaluate *Piscihepevirus* replication in the context of multiple agent infections. The nonpathogenic infection of rainbow trout with CTV proved protective against the deadly salmonid virus infectious hematopoietic necrosis virus [38]; however, the fate of CTV infections in fish compromised by infections with other agents (i.e., bacteria, viruses, parasites) remains unknown. Evaluating the outcome of salmonid hepevirus infections under such scenarios will not only better our understanding of the role these viruses play in salmonid health but undoubtedly reveal insights into the underlying mechanisms affecting hepevirus replication.

## Data Availability

Sequences utilized for analyses are available in GenBank under accession numbers as provided in Tables S1 and S2.

## Supplementary Materials

The following are available online at https://www.mdpi.com/article/10.3390/v13091730/s1, Supplemental File 1—Figure S1: Alignment of the whole genome sequences, Figure S2: Phylogenetic tree showing the genetic relationship of 193 nt region of the RNA—dependent RNA polymerase of cutthroat trout virus isolates from Canada, Figure S3: CTV-2 load in Atlantic salmon mortalities and sampled fish from the heterospecific cohabitation trial, Figure S4: Amino acid alignment showing a serine rich region within the non-structural polyprotein of hepeviruses, Table S1: Piscihepevirus isolations from salmonids in Canada from 2010 to 2017, Table S2: Information of selected hepevirus complete genome sequences used in phylogenetic analyses presented in this study, Table S3: Primer and probe sequences for RT-qPCR assays, Table S4: Experimental parameters of the conspecific cohabitation trial, Table S5: Prevalence (%) of CTV-2 in sockeye, pink, and chinook salmon mucus in conspecific cohabitation challenge, Table S6: Identification of PXXP motifs in Piscihepeviruses and Orthohepeviruses. Supplemental File 2—Analytical specificity of the western Canada assay and RT-qPCR results. Supplemental File 3—Histopathological examination results.
